# A Review of Neural Data and Modelling to Explain How a Semicircular Canal Dehiscence (SCD) Causes Enhanced VEMPs, Skull Vibration Induced Nystagmus (SVIN), and the Tullio Phenomenon

**DOI:** 10.3390/audiolres13030037

**Published:** 2023-06-02

**Authors:** Ian S. Curthoys, Christopher M. Smith, Ann M. Burgess, Julia Dlugaiczyk

**Affiliations:** 1 Vestibular Research Laboratory, School of Psychology, University of Sydney, Sydney, NSW 2006, Australia; 2 Center for Anatomy and Functional Morphology, Icahn School of Medicine at Mount Sinai, Annenberg Building, Room 12-90, 1468 Madison Ave., New York, NY 10029, USA; christopher.smith1@mssm.edu; 3 Department of Otorhinolaryngology, Head and Neck Surgery & Interdisciplinary Center of Vertigo, Balance and Ocular Motor Disorders, University Hospital Zurich (USZ), University of Zurich (UZH), CH-8091 Zürich, Switzerland

**Keywords:** SCD, dehiscence, semicircular canal, otolith, nystagmus, Tullio, SVIN, oVEMP, cVEMP, sound, vibration

## Abstract

Angular acceleration stimulation of a semicircular canal causes an increased firing rate in primary canal afferent neurons that result in nystagmus in healthy adult animals. However, increased firing rate in canal afferent neurons can also be caused by sound or vibration in patients after a semicircular canal dehiscence, and so these unusual stimuli will also cause nystagmus. The recent data and model by Iversen and Rabbitt show that sound or vibration may increase firing rate either by neural activation locked to the individual cycles of the stimulus or by slow changes in firing rate due to fluid pumping (“acoustic streaming”), which causes cupula deflection. Both mechanisms will act to increase the primary afferent firing rate and so trigger nystagmus. The primary afferent data in guinea pigs indicate that in some situations, these two mechanisms may oppose each other. This review has shown how these three clinical phenomena—skull vibration-induced nystagmus, enhanced vestibular evoked myogenic potentials, and the Tullio phenomenon—have a common tie: they are caused by the new response of semicircular canal afferent neurons to sound and vibration after a semicircular canal dehiscence.

## 1. Introduction

Air-conducted sound (ACS) and bone-conducted vibration (BCV) are now routinely used for clinical testing of vestibular function because animal studies have shown that vestibular receptors and primary afferent neurons can be activated by these stimuli (see [1,2,3,4,5] for reviews). In healthy animals with labyrinths encased in bone, activation has been demonstrated for such stimuli up to 3000 Hz for otolith afferents with irregular resting discharge, but only very low-frequency BCV (less than about 200 Hz) is effective in activating semicircular canal afferents with irregular resting discharge [6]. However, after dehiscence of the bony wall of a semicircular canal (SCD), canal neurons can, similar to otolith afferents, be activated by very high frequencies (>3000 Hz). A dehiscence is an opening, a defect, of the bony wall encasing a semicircular canal. Corresponding to those neural results, human patients with an SCD, ACS, or BCV stimuli cause a pattern of clinical auditory and vestibular symptoms, now called the third window syndrome [7]. This paper is concerned with the neural basis for the activation which causes these symptoms, which usually results in the patient showing the following characteristic results on three tests of vestibular function using BCV or ACS:

1. Enhanced vestibular evoked myogenic potentials (VEMPs), see [5,8,9,10] and
2. Skull vibration-induced nystagmus (SVIN) reviewed in [11];
3. Nystagmus and vertigo during maintained sound or vibration stimulation (the Tullio phenomenon) [12,13].

Not all patients with a CT-verified SCD show all these results, and the variability, even within a patient, has been puzzling. In this paper, we show that some of the puzzles can now be resolved thanks to recent modeling, neural recordings, and direct measurement of fluid movement in a semicircular canal after an SCD [14]. Angular acceleration stimulation of a canal causes an increased firing rate in primary vestibular afferents that result in nystagmus in healthy adult animals. However, increased firing rate can also be caused by ACS or BCV in SCD patients, and so these unusual stimuli will also cause nystagmus. ACS or BCV may increase the firing rate by either cycle-by-cycle phase-locked activation or by slow changes in firing rate due to fluid pumping, which causes cupula deflection. Both will act to increase the primary afferent firing rate and so trigger nystagmus. In some circumstances, the primary afferent data show that these two mechanisms may oppose each other. 

We first review the neural data showing how sound and vibration cause neural activation in primary afferent neurons in healthy animals (and so presumably in humans) and then address how SCD changes these neural results to explain SVIN, VEMPs, and the Tullio phenomenon.

## 2. Neural Results after SCD—History

Carey et al. provided some of the first evidence for the neural basis of these post-SCD vestibular symptoms by recording the response of single primary semicircular canal neurons in chinchillas to ACS before and after making a dehiscence in the bony wall of the anterior semicircular canal [15]. They reported that canal afferent neurons, which had been unresponsive to ACS prior to the SCD, showed strong responses after the SCD. The fluid mechanical basis for this change came from data and analysis showing that such a dehiscence changes the fluid mechanical operation of the semicircular canal and the whole labyrinth [16,17]. 

More specifically, Carey et al. demonstrated that neurons with irregular resting discharge showed cycle-by-cycle phase-locked activation by the ACS stimulus and suggested that this occurred because each compression of the endolymph caused by the sound stimulus would deflect the hair bundles of canal receptors on the crista of the canal with the SCD, and so cause phase-locked activation of irregular afferent neurons [15]. Consistent with that suggestion was the observation that this phase-locked canal response was itself locked to the stimulus presentation: starting at stimulus onset and ceasing at stimulus offset, with little or no decay or reversal of the neural response as occurs with angular acceleration stimulation [15,18]. (It should be noted that the BCV stimuli in tests of SVIN are usually of much shorter duration than angular accelerations [11]). However, after an SCD, neurons with regular resting discharge showed a very different response pattern to maintained stimuli: slow maintained changes in firing rate during an ACS stimulus but without phase-locking to individual cycles of the stimulus. Carey et al. suggested this slow response was due to the cupula being slowly deflected by sound-induced fluid flow (now called acoustic streaming) in the semicircular duct of the canal with the SCD, which was in turn caused by repeated compression of the flexible membranous semicircular duct in response to the sound stimulus [15]. Both of these responses in primary afferent firing would lead to increased canal activation and the vestibular clinical test results described above. However, some results were puzzling. There was:

1. A reversal of the slow neural activation to maintained ACS depending on frequency (see Figure 4 of [15])—so within an individual neuron, some frequencies caused excitation, and other frequencies caused inhibition, presumably indicating a reversal of endolymph flow at some frequencies;

2. Spread of activation: afferent neurons from canals other than the one with the SCD may be activated, as demonstrated by recordings of horizontal canal neural activation after anterior canal SCD (Figures 4 and 5 of [15]).

Grieser et al. modeled the acoustic streaming within a semicircular canal after dehiscence as an example of the fluid flow seen in an “impedance pump”, where repeated compressions of a flexible tube (without any valve) result in unidirectional fluid flow within that tube [19]. This is the Liebau phenomenon—a highly variable and unstable non-linear phenomenon where the direction of fluid flow depends on many physical aspects of the duct and of the stimulus, including the frequency of the compressions, the location of the compression (or SCD), and the mechanical characteristics of the duct [19,20]. The cupula would be deflected by such fluid flow activating receptors, and so causing nystagmus—the Tullio phenomenon.

Grieser et al. demonstrated that their model could explain many aspects of the data of Carey et al. and so of the Tullio phenomenon [19]. However, as later noted by Iversen et al., the Grieser model could not explain the frequency-dependent reversal of the neural response to ACS or the spread of activation to other canals without an SCD [14].

**Figure 1:**
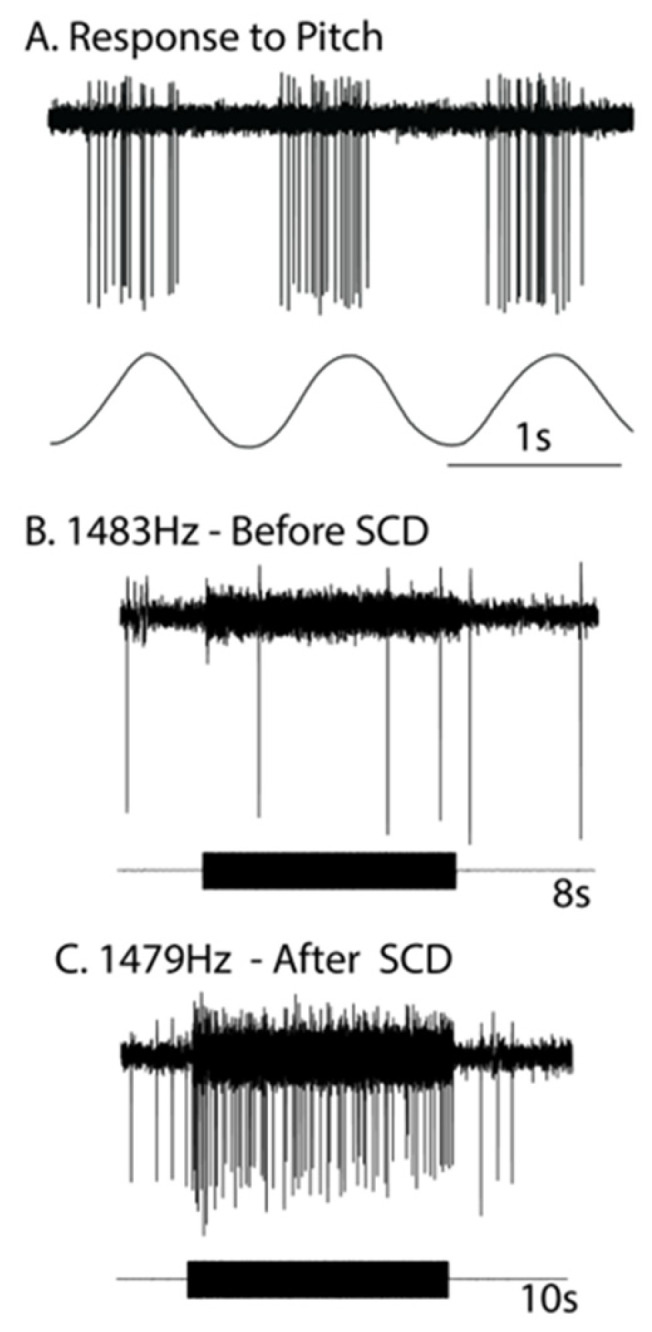
The response of the one anterior canal neuron with irregular resting discharge to high-frequency ACS (around 1480 Hz) before and after a small dehiscence was made in the bony wall of the anterior canal. (**A**) The response of the neuron (upper trace) to pitch angular acceleration (lower trace) identifies the neuron as being an anterior canal afferent. (**B**) Before SCD, an 8 s burst of 1483 Hz ACS has no effect on the resting discharge/neural response. (**C**) After SCD, a 10 s burst of 1479 Hz strongly activates the neuron. Note the abrupt onset and offset of that increased firing—locked to stimulus onset and offset—without a slow decrease in firing after the end of the stimulus. The firing during the stimulus is phase-locked. Reproduced from Curthoys, The New Vestibular Stimuli, Sound and Vibration Experimental Brain Research 2017, 235, p. 966, with permission of Springer Nature.

Curthoys investigated primary vestibular responses to sound and vibration in anesthetized guinea pigs—both in normal healthy animals and in animals where there had been an artificial dehiscence made by shaving the bony wall of the anterior semicircular canal and carrying out an extracellular recording of primary vestibular afferents to ACS and BCV before and after the SCD [2,21]. The BCV stimulator was a standard clinical Radioear B71 bone conductor cemented to the skull, and ACS was delivered by a Telephonic TDH 49 headphone. Technically, it is difficult to make a dehiscence of the bony canal whilst continuing to record from the same neuron, but that was achieved in over 70 neurons, and an example of results of the same neuron (with irregular resting discharge) recorded before and after SCD are shown in Figure 1.

Before the dehiscence, regular canal afferents were unresponsive to ACS and BCV; however, irregular semicircular canal afferents can be activated by very low frequencies of BCV (less than 200 Hz) [6] but not by higher frequencies. As explained below, that activation by low-frequency vibration is the basis of SVIN in patients with unilateral vestibular loss [22]. Apart from that exception, semicircular canal neurons in animals with normally encased bony canals show poor or absent response to the standard frequency used in clinical vestibular testing, 500 Hz BCV, even at high intensities [2,6,23]. This is true for both neurons with regular resting discharge and those with irregular resting discharge. Such a result is in sharp contrast to the response of otolithic irregular primary neurons, which show clear responses to BCV at very low intensities even before the dehiscence [2,23,24]. The response of otolithic afferents to BCV is even more sensitive after the SCD [2].

After a dehiscence, semicircular canal neurons with irregular resting discharge showed strong activation (Figure 1C) with cycle-by-cycle activation by 1479 Hz BCV. This is in sharp contrast to the absence of activation in these neurons prior to dehiscence (Figure 1B). The action potentials were locked to a particular phase angle of the BCV stimulus (or a narrow band of phase angles), similar to the phase-locking found in otolithic afferents [25]. They did not fire on every single cycle; however, the moment they fired was locked to a narrow band of phase angles of the sinusoidal stimulus [25]. This phase-locked activation was obtained at up to, in some irregular neurons, very high frequencies, even up to 4000 Hz [21].

**Figure 2:**
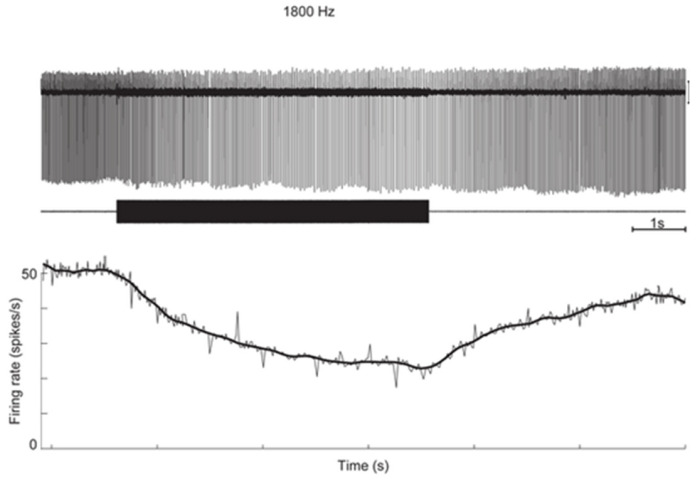
On the Iversen/Rabbitt model, after SCD, the sound-induced endolymph flow in the canal deflects the cupula, so all hair cells, including those synapsing on regular afferent neurons, change their firing rate. In the example, the regular afferent neuron (with synaptic input from type II receptors embedded in the cupula) is progressively silenced during the vibration stimulus as the cupula is deflected in an inhibitory direction by the sound-induced acoustic streaming. At the offset of the stimulus, these regular neurons progressively return to their resting discharge rate as the cupula returns to its resting position. Figure replotted from data published in [21,26].

Canal neurons with regular resting discharge did not show phase-locked activation but instead showed a slow progressive change in firing (usually a decrease in these experiments) during the maintained stimulus, followed by a return to their resting rate after the offset of that stimulus (see Figure 2). As we explain below, later modeling and evidence from recordings of toadfish primary canal afferents explains these two results.

These results in the guinea pig confirmed Carey’s main results in detail for both ACS but also showed, for the first time, that BCV stimulation elicited similar neural results to those obtained for ACS [26]. It was suggested that the guinea pig data were largely consistent with the Liebau principle, although the same inconsistencies noted by Carey et al., including the frequency-dependent reversal of neural responses, were confirmed. The present paper reviews the available neural data and shows that the apparently inconsistent data are consistent with the Iversen and Rabbitt model and results explained below. These guinea pig results were reported at the 2016 meeting of the Association for Research in Otolaryngology [26], and the following year Rabbitt and Iversen presented a new explanation of the effect of vibration on canal responses after an SCD [27], which was later formalized in detail [14]. They confirmed their model by recording single primary neurons in toadfish after SCD and by direct optical measures of fluid flow in response to BCV.

## 3. The Iversen/Rabbitt Model

**Figure 3:**
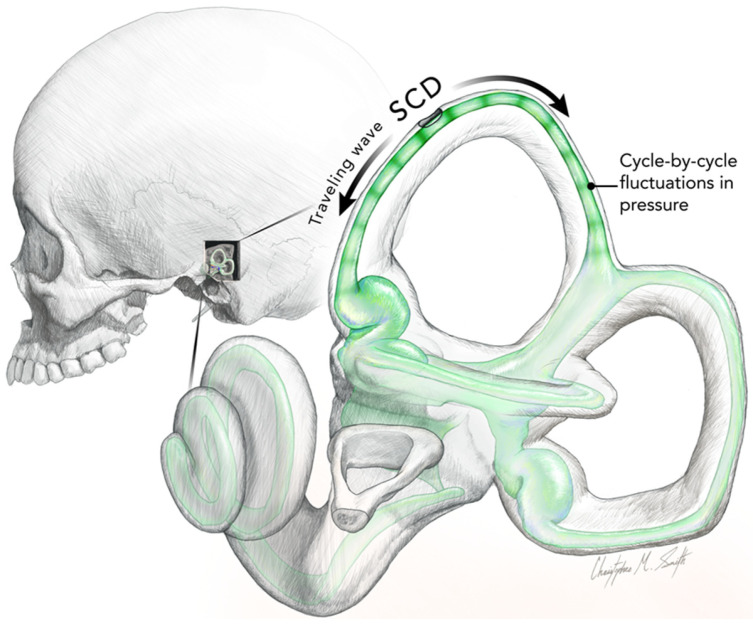
This is a representation to show the two principles of the Iversen/Rabbitt model of semicircular canal operation after an SCD. The two co-existing principles are (1) fluid streaming—traveling waves initiated at the site of the SCD and (2) cycle-by-cycle pressure changes. These concepts explain the effect of SCD on canal operation and how it generates enhanced oVEMPS, SVIN, and the Tullio phenomenon. The concepts have been demonstrated by recordings from primary canal afferents after an SCD and by the the optical measurements of fluid displacement (the acoustic streaming) in a toadfish preparation of SCD. Reproduced from Smith et al. [28] with permission of Wiley. Iversen et al. [14]) argued that the SCD relieves pressure in the perilymph at the location of the dehiscence leading to a pressure difference between endolymph and perilymph, which deforms the membranous duct and so produces a flow of endolymph. The SCD generates traveling waves that move away from the SCD along the semicircular duct in opposite directions. Such traveling waves are termed “acoustic streaming”. The first traveling wave to reach the cupula will deflect it and produce slow changes in firing in regular afferent neurons from hair cell receptors embedded in the cupula. However, in addition, there are rhythmic compressions due to the sound or vibration stimulus caused by cycle-by-cycle fluctuations in pressure within the traveling wave, and these compressions are shown here as green bands (stripes) within the duct. Such cycle-by-cycle fluctuation in pressure will deflect the hair cell receptors on the crista so, resulting in action potentials in irregular primary canal afferents phase-locked to each cycle of the stimulus frequency.

Iversen et al. modeled the acoustic streaming within the semicircular duct after SCD as being due to traveling waves originating at the SCD, traveling in both directions away from the SCD [14]. They argued that the SCD relieves pressure in the perilymph at the location of the dehiscence, leading to a pressure difference between endolymph and perilymph, which deforms the membranous duct and produces a flow of endolymph. There are cycle-by-cycle fluctuations in fluid pressure superimposed on the fluid streaming of the traveling wave. Figure 3 shows what we think is the result of these two principles in a schematic representation. The fluid streaming is faster at high frequencies, so it deflects the cupula during the stimulus and so acts to change the firing rate of regular canal neurons, as shown in Figure 2. The direction and magnitude of fluid pumping were both dependent on the stimulus frequency. The direction of that fluid flow is not fixed—it depends on many factors and changes within an experimental session. Factors such as SCD size and location, stimulus frequency, and duct impedance.

There are interesting consequences: the fluid wave is displaced in opposite directions from the location of the SCD, and as is clear from the Figure 3, the differences in length will determine which one of the waves is effective in displacing the cupula. In this figure, the distance from the SCD to the crista is shorter than the long distance from the SCD around the common crus. In this case, we would expect the acoustic streaming of the shorter arm to be effective in deflecting the cupula. At one location, the two waves will have equal distances, and so acoustic streaming will be canceled, although the cycle-by-cycle compression will occur. That may explain why some primary afferent neurons show cycle-by-cycle phase-locked activation but without any evidence of a systematic change in cupular displacement.

This fluid displacement caused by ACS or BCV will displace the cupula and so activate the semicircular canal in a manner equivalent to constant angular acceleration, and so in human patients, it would cause nystagmus and a sensation of rotation. At stimulus offset, the acoustic streaming ceases, allowing the cupula to return to its resting position by virtue of the cupula elastic restoring force [29], and as it does so, the neural firing rate will return to its resting discharge rate, as shown in Figure 2.

These results suggest that in some cases of the Tullio phenomenon, there may be some after nystagmus due to that slow cupula return, although this would only be apparent after prolonged stimulation, which is unusual in tests of the Tullio phenomenon. In many cases, the ACS and BCV stimuli are of short duration because the vertigo they produce is unpleasant for the patient. On the Iversen/Rabbitt model, the cycle-by-cycle phase-locked neural activation was attributed to the cycle-by-cycle pressure variations caused by the BCV (or ACS) stimulus, which have clearly been shown to be effective hair cell stimuli in primary otolith and canal afferents of healthy guinea pigs [14,25]. Each cycle of the sound or vibration stimulus deflects the hair bundles of canal receptors and so results in the cycle-by-cycle phase-locked activation of primary afferents.

**Figure 4:**
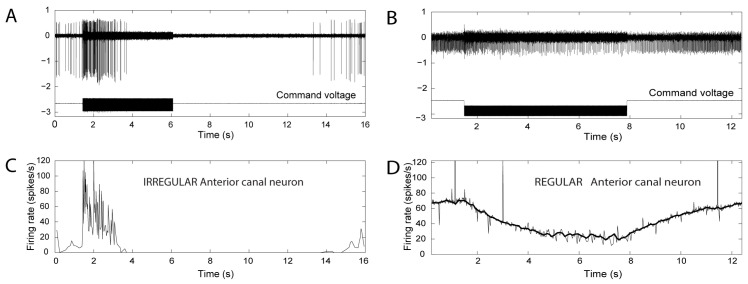
After SCD, if acoustic streaming deflects the cupula in an inhibitory direction, then all the receptor hair cells and afferents from that canal will be progressively silenced. Consequently, maintained sound or vibration stimulation will act to silence the cycle-by-cycle phase-locked activation of irregular afferents. After the cessation of the sound, the (silenced) irregular afferent gradually resumes its resting activity (**A**,**C**) as the cupula presumably slowly returns to its usual upright position because of the elastic restoring force of the cupula [29]. As the cupula returns, it removes the inhibitory drive on the regular (**B**,**D**) and irregular afferents, which return to their resting discharge rate. These two co-existing mechanisms—cycle-by-cycle activation and fluid flow—can work together, or they can oppose each other as these data suggest. Figure replotted from data published in [21,26].

The dual mechanisms of the Iversen/Rabbitt model extend the original ideas of Carey et al. [15] and explain a number of aspects of the data:

1. Activation may reverse to inhibition at particular frequencies, as Carey showed (see Figure 5 of Carey et al., 2004 [15]), depending on SCD location and stimulus frequency;
2. There can be a spread of activation of canals other than the canal with the SCD-Figures 4 and 5 of [15] show the excitation of a horizontal canal neuron after dehiscence of the anterior canal.

Importantly these two mechanisms—brief waves of compression and slow fluid flow co-exist after the SCD, and we suggest that some apparently inconsistent neural results are due to the interaction of these two mechanisms. Some irregular neurons displayed a characteristic but unusual response pattern: at high frequencies, they were strongly activated at stimulus onset and then silenced within a short time and only returned to their resting discharge rate long after the stimulus ceased (Figure 4). Initially, this appears to be a rapid frequency-dependent adaptation and appears to be inconsistent with the Iversen/Rabbitt model.

So these data, instead of appearing to be inconsistent with the Iversen model, uncover a prediction of that model and strengthen it by showing that in particular circumstances, the neural result is the likely outcome of the interaction between the two basic mechanisms of the Iversen/Rabbitt model. At very high frequencies, this decline in firing (Figure 4) took place almost immediately, whilst at lower frequencies, this silencing took longer, and, in some cases, there was no silencing at all. We now suggest that this pattern of response shown in Figure 4 and exhibited by many irregular canal afferents was due to the interaction of the cycle-by-cycle activation and the fluid streaming induced by the SCD.

Can phase-locked activation and acoustic streaming be dissociated? Many irregular afferents show maintained firing during the entire BCV stimulus without any change, which could be attributed to cupula deflection. This may occur if the two traveling waves cancel. Dissociation may also occur in cases of patients with very large SCD. Such patients have enhanced VEMPs [30], but some do not show the Tullio phenomenon. This is possibly because the dura herniates into the canal, effectively blocking the duct and so blocking the acoustic streaming, which deflects the cupula and so causes nystagmus and vertigo whilst allowing access to the cycle-by-cycle compression and activation [31]. Consequently, the enhanced VEMPs and SVIN may occur without Tullio nystagmus.

## 4. VEMPs

**Figure 5:**
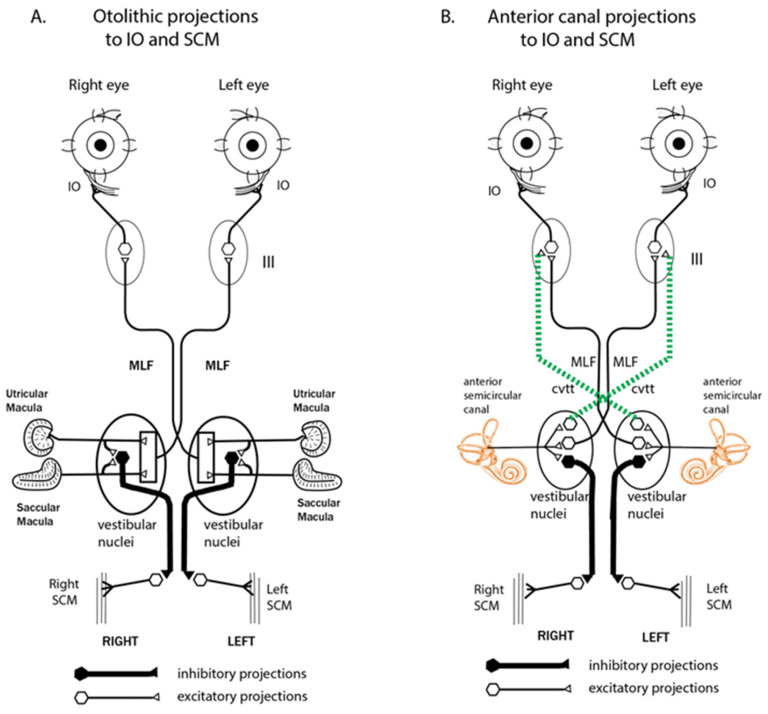
Schematic diagrams of the otolithic projections to the inferior oblique and sternocleidomastoid muscle (IO and SCM) are shown in (**A**). Panel (**B**) on the right shows the analogous projections of the anterior semicircular canal neurons to the inferior oblique eye muscles (IO) and sternocleidomastoid neck muscles (SCM) [35]. Stimulation in animals with intact labyrinths causes the neural connections shown on the left panel to be activated. However, after an SCD, the anterior semicircular canals, which project to contralateral inferior oblique and ipsilateral SCM, are also activated by sound and vibration, in addition to the otolithic projections, so the neural projections on the right (dashed lines) come into play, since these previously unresponsive canal afferents are now activated by ACS or BCV. It appears that it is this combination of otolithic and canal afferent activation which, in part, results in the enhanced oVEMP and cVEMP responses after SCD. Reproduced from Curthoys, The New Vestibular Stimuli, Sound and Vibration Experimental Brain Research 2017, 235, p. 968, with permission of Springer Nature.

Irregular otolithic afferents arising from the striola of the utricular macula [32,33] are activated at low thresholds by ACS and BCV. This activation projects via the vestibular nuclei to the contralateral inferior oblique (IO) eye muscle [34] and the ipsilateral SCM [35] (Figure 5).

The anterior canal neurons will be enhanced after SCD and so provide additional drive in addition to the otolithic afferents. The enhanced oVEMP and cVEMP responses in SCD patients [9,30,36,37] are probably due to the demonstrated projection of anterior canal afferents to the inferior oblique muscles [35] for the oVEMP and the projection of anterior canal neurons to the SCM for the cVEMP. So the dehiscence of the anterior canal acts to enhance VEMP responses by virtue of the convergence of the anterior canal and otolith neurons [38]. However, in addition, the dehiscence allows a direct pathway of compression waves to the labyrinthine fluid and so activates otolithic afferents resulting in large oVEMPs and low thresholds. This more direct pathway may be the reason that oVEMPs to BCV stimulation at the vertex (Cz) are large in SCD patients whereas, in healthy subjects, they are relatively small [30].

## 5. Skull Vibration-Induced Nystagmus (SVIN)

SVIN is the nystagmus induced by 100 Hz vibration applied to either mastoid in patients with asymmetric vestibular function [11,39]. A recent paper explained the basis of skull vibration-induced nystagmus (SVIN) in patients with total unilateral vestibular loss (uVL) in terms of neural responses of irregular semicircular canal neurons in guinea pigs after an SCD [22]. In animals with intact canals encased in bone (as normal), low-frequency ACS and BCV (less than about 200 Hz) activate canal neurons with irregular resting discharge in a cycle-by-cycle phase-locked fashion. Frequencies of 500 Hz and above are ineffective in such canal neuronal activation [6] and in human patients [40]. In healthy people, the afferent neural input from simultaneous activation of both labyrinths by mastoid stimulation will cancel at the vestibular nuclei, so there will be no nystagmus. However, after uVL, 100 Hz vibration of either mastoid activates semicircular canal neurons, but in a patient with uVL, there is no cancellation, so the 100 Hz activation will cause time-locked nystagmus (SVIN) with time-locked horizontal and torsional components, beating away from the affected ear [22]. The simultaneous activation of the anterior and posterior canals of the remaining labyrinth will cancel each other, so there will be little or no vertical component [11,22].

Patients with total uVL are uncommon, and it is much more usual to test patients with a partial unilateral loss or an SCD, and in such cases, the pattern of nystagmus is not as clear cut as for uVL patients [40,41,42]. Recordings of SVIN in patients with CT-verified SCD show that the majority of these patients show nystagmus with quick phases directed towards the ear with the SCD [40,42,43] but with a strong vertical component, unlike the SVIN after uVD. However, some SCD patients show the opposite direction of nystagmus. It appears that one of the reasons for this directional difference may be the exact location of that dehiscence in particular patients, but other factors (e.g., stimulus frequency, stimulus location) can affect these mechanisms. The strong vertical component may be due to the direct activation of the anterior canal not being canceled by the activation of the posterior canal.

Elsewhere we have argued that the SVIN nystagmus induced by BCV is a variant of the standard Tullio phenomenon, which originally was described in relation to air-conducted sound [12,43]. That SVIN nystagmus is unusual in that it starts immediately at stimulus onset and ceases at stimulus offset with no after nystagmus and so corresponds to the tight time-locked firing of canal afferents to BCV.

## 6. Discussion

### Implications for Diagnosis

From the point of view of the clinician, how is it possible to get confirmation of SCD from these functional test results, which are apparently so variable? There are two tests that we think help in identifying an SCD:

(1) Test with ACS or BCV at high frequencies (500 or 750 Hz). The standard SVIN stimulus frequency of 100 Hz is almost ideal for activating irregular semicircular canal neurons if the duct is encased in bone [11]. However, the neural data from recordings of primary afferents after SCD showed that irregular canal neurons could be activated up to very high frequencies of ACS or BCV [21]. So Curthoys urged his colleague Georges Dumas to try high frequencies in SCD patients, and he did so and found clear SVIN response at high frequencies (e.g., 500 and 750 Hz) [40], far above the effective frequencies for total or partial unilateral vestibular loss. In human patients, the presence of SVIN at high frequencies (such as 500 or 750 Hz) is an indicator of SCD [43,44]);
(2) It is valuable to test different BCV stimulus locations: BCV stimulation at the vertex of the skull in healthy people causes small or absent oVEMPs [30] and little or no nystagmus response [43]. However, in SCD patients, there is a very clear SVIN nystagmus generated by such vertex stimulation [43]. This is in agreement with the fact that vertex (Cz) BCV stimulation causes small or absent oVEMPs in healthy people, but in SCD patients there is a clear oVEMP n10 [30].

## 7. Conclusions

This review has shown how these three clinical phenomena, SVIN, VEMPs, and Tullio phenomenon, have common ties: they are caused by the response of canal afferent neurons to sound and vibration after an SCD.

In summary: the variable character of responses of SCD patients to BCV, which initially appears to be inconsistent with neural results, in fact, is consistent with the unusual fluid flow mechanisms initiated by the SCD. Once the labyrinth is opened, then the fluid flow that is generated by vibration or sound is highly unstable. It can reverse direction depending on where the actual vibration is located and on the frequency of the vibration stimulation and duct characteristics. This instability is in contrast to the direction of SVIN in unilateral vestibular loss, where the nystagmus direction reliably identifies the affected ear since the nystagmus beats away from the affected ear. The neural results of BCV after SCD from guinea pigs confirm earlier results from Carey et al. [15]). While initially puzzling, these results are indeed consistent with the predictions from the Iversen/Rabbitt model of traveling waves and stimulus-locked compressive waves in endolymph after SCD. Table 1 is a summary of the major clinical observations after SCD and their probable neural bases.

## Figures and Tables

**Table 1:** Neural evidence explaining vestibular effects of unilateral SCD.

Clinical Observation after Unilateral SCD	References for Clinical Observations	Probable Neural Cause	References for Probable Neural Cause
Enhanced oVEMP to 500 Hz or clicks (ACS or BCV)	[30]	Cycle-by-cycle phase-locked activation of irregular canal afferent neurons to ACS and BCV after SCD to 500 Hz	[2,3,8,15,23,24,25,33,45,46,47,48,49]
Enhanced oVEMP to very high-frequency ACS or BCV (4000 Hz)	[48,50]	Cycle-by-cycle phase-locked activation of irregular canal afferent neurons to ACS and BCV to very high frequencies after SCD	[21,25,45]
Nystagmus and vertigo in response to maintained ACS or BCV (Tullio phenomenon). The direction of quick phases usually towards the ear with the SCD but may be opposite (determined by many factors, such as skull location of BCV stimulation (see text))	[9,51,52,53,54]	Acoustic streaming of endolymph generated by repeated compression of the semicircular duct. The “traveling wave hypothesis” of Iversen and Rabbitt. Factors such as location of the SCD (proximity to ampulla), stimulus frequency, BCV stimulator location, and membranous labyrinth impedance determine acoustic streaming. (Non-linear process). Complemented by a cycle-by-cycle phase-locked afferent activation	[14,15,19,26]
Skull Vibration Induced Nystagmus (SVIN). The direction of quick phases is usually towards the ear with the SCD but may be opposite (determined by many factors, such as skull location of BCV stimulation (see text)). Anterior canal SCD generates vertical nystagmus	[11,40,41,42,55,56]	Probably mainly due to cycle-by-cycle phase-locked activation. The direction of the nystagmus is determined by factors such as the location of the SCD (proximity to ampulla), stimulus frequency, BCV stimulator location, and membranous labyrinth impedance, which determine acoustic streaming. (Non-linear process).	[14,15]
SVIN components in canal planes different from the canal with the SCD (e.g., horizontal components after anterior canal dehiscence).	[15]	The cycle-by-cycle compression is not restricted to only the canal with the SCD, so afferents from other canals show phase-locked neural responses. (Figures 4 and 5 of [15]) Iversen et al.). Also, otolithic afferents show enhanced responses (Curthoys 2017 [2])	[14,15]

## Data Availability

Not applicable.

## Abbreviations

ACS	Air-conducted sound
BCV	bone-conducted vibration
IO	inferior oblique eye muscle
QP:	quick phases
SCD	semicircular canal dehiscence
SCM	sternocleidomastoid neck muscle
SPV	slow phase eye velocity
SVIN	skull vibration-induced nystagmus
TUVL	total unilateral vestibular loss
VEMP	vestibular evoked myogenic potential
VN	vestibular nuclei

## References

[1] Curthoys I.S. (2010). A critical review of the neurophysiological evidence underlying clinical vestibular testing using sound, vibration and galvanic stimuli. Clin. Neurophysiol..

[2] Curthoys I.S. (2017). The new vestibular stimuli: Sound and vibration-anatomical, physiological and clinical evidence. Exp. Brain Res..

[3] Curthoys I.S., Vulovic V., Burgess A.M., Manzari L., Sokolic L., Pogson J., Robins M., Mezey L.E., Goonetilleke S., Cornell E.D. (2014). Neural basis of new clinical vestibular tests: Otolithic neural responses to sound and vibration. Clin. Exp. Pharmacol. Physiol..

[4] McCaslin D.L., Jacobson G.P. (2009). Current role of the videonystagmography examination in the context of the multidimensional balance function test battery. Semin. Hear..

[5] Rosengren S.M., Colebatch J.G. (2018). The contributions of vestibular evoked myogenic potentials and acoustic vestibular stimulation to our understanding of the vestibular system. Front. Neurol..

[6] Dlugaiczyk J., Burgess A.M., Curthoys I.S. (2020). Activation of Guinea Pig Irregular Semicircular Canal Afferents by 100 Hz Vibration: Clinical Implications for Vibration-induced Nystagmus and Vestibular-evoked Myogenic Potentials. Otol. Neurotol..

[7] Wackym P.A., Agrawal Y., Ikezono T., Balaban C.D. (2021). Editorial: Third Window Syndrome. Front. Neurol..

[8] Curthoys I.S., Dlugaiczyk J. (2020). Physiology, clinical evidence and diagnostic relevance of sound-induced and vibration-induced vestibular stimulation. Curr. Opin. Neurol..

[9] Watson S.R., Halmagyi G.M., Colebatch J.G. (2000). Vestibular hypersensitivity to sound (*Tullio phenomenon*): Structural and functional assessment. Neurology.

[10] Bronstein A.M., Faldon M., Rothwell J., Gresty M.A., Colebatch J., Ludman H. (1995). Clinical and electrophysiological findings in the Tullio phenomenon. Acta Otolaryngol. Suppl..

[11] Dumas G., Curthoys I.S., Lion A., Perrin P., Schmerber S. (2017). The skull vibration-induced nystagmus test of vestibular function-a review. Front. Neurol..

[12] Tullio P. (1928). L’orecchio.

[13] Halmagyi G.M., Curthoys I.S., Colebatch J.G., Aw S.T. (2005). Vestibular responses to sound. Ann. N. Y. Acad. Sci..

[14] Iversen M.M., Zhu H., Zhou W., Della Santina C.C., Carey J.P., Rabbitt R.D. (2018). Sound abnormally stimulates the vestibular system in canal dehiscence syndrome by generating pathological fluid-mechanical waves. Sci. Rep..

[15] Carey J.P., Hirvonen T.P., Hullar T.E., Minor L.B. (2004). Acoustic responses of vestibular afferents in a model of superior canal dehiscence. Otol. Neurotol..

[16] Rosowski J.J., Songer J.E., Nakajima H.H., Brinsko K.M., Merchant S.N. (2004). Clinical, experimental, and theoretical investigations of the effect of superior semicircular canal dehiscence on hearing mechanisms. Otol. Neurotol..

[17] Songer J.E., Rosowski J.J. (2005). The effect of superior canal dehiscence on cochlear potential in response to air-conducted stimuli in chinchilla. Hear. Res..

[18] Goldberg J.M., Fernandez C. (1971). Physiology of peripheral neurons innervating semicircular canals of the squirrel monkey. I. Resting discharge and response to constant angular accelerations. J. Neurophysiol..

[19] Grieser B.J., Kleiser L., Obrist D. (2016). Identifying Mechanisms Behind the Tullio Phenomenon: A Computational Study Based on First Principles. Jaro J. Assoc. Res. Otolaryngol..

[20] Liebau G. (1954). Uber ein ventilloses pumpprinzip. Naturwissenschaften.

[21] Dlugaiczyk J., Burgess A.M., Goonetilleke S.C., Sokolic L., Curthoys I.S. (2019). Superior Canal Dehiscence syndrome: Relating clinical findings with vestibular neural responses from a guinea pig model. Otol. Neurotol..

[22] Curthoys I.S. (2021). The Neural Basis of Skull Vibration Induced Nystagmus (SVIN). Audiol. Res..

[23] Curthoys I.S., Kim J., McPhedran S.K., Camp A.J. (2006). Bone conducted vibration selectively activates irregular primary otolithic vestibular neurons in the guinea pig. Exp. Brain Res..

[24] Curthoys I.S., Vulovic V. (2011). Vestibular primary afferent responses to sound and vibration in the guinea pig. Exp. Brain Res..

[25] Curthoys I.S., Burgess A.M., Goonetilleke S.C. (2019). Phase-locking of irregular guinea pig primary vestibular afferents to high frequency (>250 Hz) sound and vibration. Hear. Res..

[26] Curthoys I.S., Grant J.W. (2016). In what way is an air conducted sound an otolithic stimulus?. Assoc. Res. Otolaryngol. Abstr..

[27] Rabbitt R.D., Iversen M.M. (2017). Elasto-hydrodynamic Model of the Deformable Semicircular Canals. Assoc. Res. Otolaryngol. Abstr..

[28] Smith C.M., Curthoys I.S., Mukherjee P., Wong C., Laitman J.T. (2022). Three-dimensional visualization of the human membranous labyrinth: The membrana limitans and its role in vestibular form. Anat. Rec..

[29] Rabbitt R.D., Breneman K.D., King C., Yamauchi A.M., Boyle R., Highstein S.M. (2009). Dynamic Displacement of Normal and Detached Semicircular Canal Cupula. Jaro J. Assoc. Res. Otolaryngol..

[30] Manzari L., Burgess A.M., McGarvie L.A., Curthoys I.S. (2012). Ocular and cervical vestibular evoked myogenic potentials to 500 Hz fz bone-conducted vibration in superior semicircular canal dehiscence. Ear Hear..

[31] Manzari L., Burgess A.M., MacDougall H.G., Curthoys I.S. (2011). Enhanced otolithic function in semicircular canal dehiscence. Acta Otolaryngol..

[32] Fernandez C., Lysakowski A., Goldberg J.M. (1995). Hair-cell counts and afferent innervation patterns in the cristae ampullares of the squirrel monkey with a comparison to the chinchilla. J. Neurophysiol..

[33] Curthoys I.S., Vulovic V., Burgess A.M., Sokolic L., Goonetilleke S.C. (2016). The response of guinea pig primary utricular and saccular irregular neurons to bone-conducted vibration (BCV) and air-conducted, sound (ACS). Hear. Res..

[34] Suzuki J.I., Tokumasu K., Goto K. (1969). Eye movements from single utricular nerve stimulation in the cat. Acta Otolaryngol..

[35] Uchino Y., Kushiro K. (2011). Differences between otolith- and semicircular canal-activated neural circuitry in the vestibular system. Neurosci. Res..

[36] Streubel S.O., Cremer P.D., Carey J.P., Weg N., Minor L.B. (2001). Vestibular-evoked myogenic potentials in the diagnosis of superior canal dehiscence syndrome. Acta Otolaryngol. Suppl..

[37] Welgampola M.S., Myrie O.A., Minor L.B., Carey J.P. (2008). Vestibular-evoked myogenic potential thresholds normalize on plugging superior canal dehiscence. Neurology.

[38] Curthoys I.S., Grant J.W., Burgess A.M., Pastras C.J., Brown D.J., Manzari L. (2018). Otolithic Receptor Mechanisms for Vestibular-Evoked Myogenic Potentials: A Review. Front. Neurol..

[39] Dumas G., Perrin P., Schmerber S. (2008). Nystagmus induced by high frequency vibrations of the skull in total unilateral peripheral vestibular lesions. Acta Otolaryngol..

[40] Dumas G., Tan H., Dumas L., Perrin P., Lion A., Schmerber S. (2019). Skull vibration induced nystagmus in patients with superior semicircular canal dehiscence. Eur. Ann. Otorhinolaryngol.-Head Neck Dis..

[41] Dumas G., Lion A., Karkas A., Perrin P., Perottino F., Schmerber S. (2014). Skull vibration-induced nystagmus test in unilateral superior canal dehiscence and otosclerosis: A vestibular Weber test. Acta Otolaryngol..

[42] Schmerber S., Dumas G., Perrin P. (2008). Anterior semicircular canal dehiscence and cranial vibration-induced nystagmus test. Otol. Neurotol..

[43] Dumas G., Curthoys I.S., Catellucci A., Perrin P., Dumas L., Schmerber S. (2023). A bone conducted Tullio phenomenon—A bridge to understand Skull Vibration Induced Nystagmus in Superior Canal Dehiscence. Front. Neurol..

[44] Dumas G., Quatre R., Schmerber S. (2021). How to do and why perform the skull vibration-induced nystagmus test. Eur. Ann. Otorhinolaryngol. Head Neck Dis..

[45] Curthoys I.S., Vulovic V., Sokolic L., Pogson J., Burgess A., Grant W. (2014). The frequency responses of irregular primary utricular afferent neurons to bone conducted vibration (BCV) and air-conducted sound (ACS). J. Vestib. Res..

[46] Curthoys I.S., Vulovic V., Sokolic L., Pogson J., Robins M., Burgess A. (2013). The neural basis of clinical vestibular responses to bone conducted vibration (BCV) and air-conducted sound (ACS). ARO Abstr..

[47] Curthoys I.S., MacDougall H.G., Vidal P.P., de Waele C. (2017). Sustained and transient vestibular systems: A physiological basis for interpreting vestibular function. Front. Neurol..

[48] Curthoys I.S., Manzari L. (2020). A simple specific functional test for SCD: VEMPs to high frequency (4000Hz) stimuli-their origin and explanation. Front. Neurol..

[49] Curthoys I.S., Vulovic V., Sokolic L., Pogson J., Burgess A.M. (2012). Irregular primary otolith afferents from the guinea pig utricular and saccular maculae respond to both bone conducted vibration and to air conducted sound. Brain Res. Bull..

[50] Manzari L., Burgess A.M., McGarvie L.A., Curthoys I.S. (2013). An Indicator of Probable Semicircular Canal Dehiscence: Ocular Vestibular Evoked Myogenic Potentials to High Frequencies. Otolaryngol. Head Neck Surg..

[51] Colebatch J.G., Day B.L., Bronstein A.M., Davies R.A., Gresty M.A., Luxon L.M., Rothwell J.C. (1998). Vestibular hypersensitivity to clicks is characteristic of the Tullio phenomenon. J. Neurol. Neurosurg. Psychiatry.

[52] Colebatch J.G., Rothwell J.C., Bronstein A., Ludman H. (1994). Click-evoked vestibular activation in the Tullio phenomenon. J. Neurol. Neurosurg. Psychiatry.

[53] Kaski D., Bronstein A.M. (2016). Patients with vestibular loss, tullio phenomenon, and pressure-induced nystagmus: Vestibular atelectasis?. Otol. Neurotol..

[54] Kaski D., Davies R., Luxon L., Bronstein A.M., Rudge P. (2012). The Tullio phenomenon: A neurologically neglected presentation. J. Neurol..

[55] Fabre C., Tan H.Y., Dumas G., Giraud L., Perrin P., Schmerber S. (2021). Skull Vibration Induced Nystagmus Test: Correlations with Semicircular Canal and Otolith Asymmetries. Audiol. Res..

[56] White J.A., Hughes G.B., Ruggieri P.N. (2007). Vibration-induced nystagmus as an office procedure for the diagnosis of superior semicircular canal dehiscence. Otol. Neurotol..

